# An exploration of risk for recurrent falls in two geriatric care settings

**DOI:** 10.1186/1471-2318-13-106

**Published:** 2013-10-10

**Authors:** Humeira Tariq, Marita Kloseck, Richard G Crilly, Iris Gutmanis, Maggie Gibson

**Affiliations:** 1 Graduate Program in Health and Rehabilitation Sciences, Faculty of Health Sciences, Western University, Ontario, Canada; 2 School of Health Studies, Faculty of Health Sciences, Western University, Ontario, Canada; 3 Division of Geriatric Medicine, Schulich School of Medicine, Western University, Ontario, Canada; 4 Specialized Geriatric Services, Parkwood Hospital, St. Joseph’s Health Care London, Ontario, Canada; 5 Veterans’ Care Program, Parkwood Hospital, St. Joseph’s Health Care London, Ontario, Canada

**Keywords:** Repeat falls, Geriatric rehabilitation, Long stay veterans’ unit, Quality assurance

## Abstract

**Background:**

Fall events were examined in two distinct geriatric populations to identify factors associated with repeat fallers, and to examine whether patients who use gait aids, specifically a walker, were more likely to experience repeat falls. Each unit already had a generic program for falls prevention in place.

**Methods:**

Secondary data analysis was conducted on information collected during the pilot testing of a new quality assurance Incident Reporting Tool between October 2006 and September 2008. The study settings included an in-patient geriatric rehabilitation unit (GRU) and a long stay veterans’ unit (LSVU) in a rehabilitation and long-stay hospital in Ontario. Participants were two hundred and twenty three individuals, aged 65 years or older on these two units, who experienced one or more fall incidents during the study period.

**Results:**

Logistic regression analyses showed that on the GRU age was significantly associated with repeat falls. On the LSVU first falls in the morning or late evening were associated with repeat falling. Walker as a gait aid listed at time of first fall was not associated with repeat falls.

**Conclusions:**

This study suggests that different intervention may be necessary in different geriatric settings to identify, for secondary prevention, certain individuals for which the generic programs prove inadequate. Information collection with a specific focus on the issue of repeat falls may be necessary for greater insight.

## Background

Falls and fall-related injuries are a common and serious problem among older adults as such events can result in disability, chronic pain, loss of independence, a reduced quality of life, and in severe cases, even death [1]. Falls among adults aged 65 or more years are three times more frequent for those who live in institutional settings than those living in the community [2]. In Canada approximately 7.4% of older adults who are 65 or more years of age live in institutional settings yet this group experiences 21% of all fall-related hospitalizations. Also, more than 75% of all fall-related injuries for this group were to a major joint, femur, pelvis, hip or thigh [3].

Many factors are associated with falls. This high propensity for falls and fall-related injury in the elderly, especially in institutional settings, is likely related to a higher prevalence of co-morbid diseases as well as physiological and cognitive decline associated with aging. Falls in institutions result from a complex interaction between individual specific or intrinsic risk factors, extrinsic factors pertaining to the physical environment, and the person’s own risk taking behaviour [4].

Another important aspect to be considered is recurrent falls. Studies have found that approximately 50% of all long-term care (LTC) home residents fall each year, and of these, 40% fall twice or more each year [5,6]. Risk factors associated with recurrent falls are similar to those of single falls [7] but because recurrent fallers are more likely to experience injury from repeated episodes, they constitute an important group to target for preventive efforts [8].

Walkers, canes and other mobility aids can improve balance and gait safety among older adults. However, the use of such devices is also associated with an increased risk of falling [9]. Walkers that are the wrong size, are used improperly, or are in a poor state of repair can contribute to unsafe mobility [10]. Among LTC home residents, use of a cane/walker was found to be independently associated with falling [6,11]. However, few Canadian studies have explored the impact of walkers on the risk of recurrent falling and injury [12].

Falls are a major problem in geriatric care and the most commonly reported patient/resident safety incidents [13]. Health care for older adults can be offered in a wide range of settings including geriatric rehabilitation units, psycho-geriatric wards, and LTC facilities. Fall incidence rates vary by health care setting, likely reflecting differences in patient population characteristics and activity patterns [14].

All health care settings are charged with keeping patients/residents safe and implementing fall prevention/reduction strategies. In the report “Prevention of Falls in Long-Term Care Facilities” [15], the Canadian Task Force on Preventive Health Care recommends comprehensive and individualized assessment of the broad range of extrinsic and intrinsic risk factors for falls for all persons at time of admission to LTC, following which multifactorial intervention programs, tailored to reduce extrinsic and intrinsic fall risk factors for each resident, should be implemented. Residents should also be reassessed after a fall, and interventions modified to address identified risk factors. The above recommendations are supported by the Registered Nurses’ Association of Ontario (RNAO) in their Best Practice Guidelines for Prevention of Falls and Fall Injuries in the Older Adult, (2005) [13] and by the Ontario Ministry of Health and Long-term Care in their Long-Term Care Homes Act, (2007) [16]. Most health care facilities have implemented fall risk reduction programs. It is impossible to prevent all falls and some still occur. Some are due to random and unpredictable circumstances and events and thus extremely challenging to prevent. If, however, a patient is experiencing recurrent falling this suggests a more individualized approach is needed. It would be useful, however, if patterns that predict recurrent fallers could be discerned and acted upon to prevent the recurrent falls. The type of information and how it is collected are critical if patterns are to be detected and acted upon. It is also likely that data will need to be collected on non-fallers and single fallers for comparative purposes.

The purpose of this exploratory pilot study was to examine whether factors associated with repeat falls can be identified and if they differ across health care settings or if there are common issues independent of setting. For this reason we have deliberately selected two quite disparate care settings for elderly people.

## Methods

The study was conducted at a hospital in a major urban centre in Ontario which provides both geriatric rehabilitation and long term care. This hospital has a number of units specific to client needs. For this study fall events on two units were examined. The Geriatric Rehabilitation Unit (GRU), a 50 bed unit with an average occupancy of 93.0%, provides rehabilitation services to older patients, a large proportion of whom have musculoskeletal (orthopaedic) health issues. Many on this unit are aged, frail and have multiple co-morbidities. The majority of patients are discharged home but a minority go to long term care institutions. Fall events were also examined on a long-stay Veterans’ Unit (LSVU) which serves the needs of older veterans, mostly men, who require residential care. The unit has 80 beds with an average occupancy of 80.9%. Residents are for the most part mobile, rarely confined to bed and usually remain for the rest of their lives.

In order to develop a better understanding of adverse events including falls, a new incident reporting measure that provided more fall event descriptors, was developed in 2006. During the pilot testing of this new tool, information was collected on paper and then entered into an electronic database. A secondary data analysis was conducted using this database to examine the type and frequency of falls and fall-related injuries among older adults receiving care on the two study units.

Past fall frequency information suggested that two years of data would be needed to compare fall events between the two groups. Thus, data on all patients who were on these two units between October 2006 and September 2008 and who had sustained a fall were extracted from the overall incident database for analysis. Variables included a patient identifier, unit, age, time of day of first fall, reported use of mobility aid, and whether or not the person sustained an injury. Gender was not available from the incident database but was added based on information in the client record. In addition, length of stay for all patients who had been on these two units during the study period was collected.

### Statistical analyses

First, the information captured in the study database was examined to make sure that it included fall events specific to the research question. Then, repeat fallers were identified and factors associated with the first fall event were examined. Pearson Chi-Square tests were used to identify significant differences between the two units with regard to gender, injury sustained at time of fall, gait aid listed at time of fall, and time of day of fall. T-tests were used to examine unit differences by age and number of falls.

Following this, multivariate binary logistic regression analysis was conducted to identify factors associated with repeat fallers. Anyone who fell more than one time during their length of stay, irrespective of the duration of stay, was classified as a repeat faller. Categorical variables with more than two categories were re-coded as dummy variables. Variables were entered sequentially in blocks (Step 1: use of gait aid; Step 2: hospital unit; Step 3: age and gender; Step 4: injury and time of day of first fall). The Hosmer and Lemeshow Chi-square Test of Goodness of Fit was used to test the overall fit of each regression model [17]. A similar approach was used to build a model for each unit separately. Statistical tests were based on a two-tailed level of significance, with an alpha value of 0.05. The analysis was conducted using SPSS version 18.

The study was approved by The University of Western Ontario Review Board for Health Science Research Involving Human Subjects.

## Results

The original data file with 590 fall events included falls that happened to those who were less than 65 years of age (22 events) and 10 near-misses (defined as an event or incident that could have resulted in a fall, but did not, either by chance or timely intervention). These fall events were excluded from the study data file. In addition, data were missing for an additional 13 fall events. The final data file used for this study included descriptors of 545 fall events that occurred to 223 people.

The characteristics of everyone who had received care on either the GRU or the LSVU during the study period were reviewed. Twice as many females (F) were admitted to the GRU than males (M) (F: 526 or 66.8% vs. M: 261 or 33.2%). On the LSVU, there were many more males than females, as expected given the population served (older war veterans) (M: 96 or 91.4% vs. F: 9 or 8.6%). Residents on the LSVU had a much longer mean length of stay than patients on the GRU (LSVU: 714 days; GRU: 40 days). The mean age of residents on the LSVU was greater than that of patients on the GRU (LSVU: 87.3 years; GRU: 81.4 years). As well, the overall fall rate on the GRU during the study period was 7.7 falls per 1,000 patient days while the overall fall rate on the LSVU during the study period was 6.1 falls per 1,000 patient days.

As seen in Table 1, LSVU fallers were significantly older than those who fell while on the GRU (87.4 vs. 82.0 years, T = −5.51, p < 0.000), probably reflecting the differing age composition of the units. As well, more of the fallers on the GRU were using a walker at the time of the first fall (GRU: 65.5%; LSVU: 37.2%) than fallers on the LSVU. It is also notable that on the LSVU single fall incidents were recorded for only 29.5% of the fallers when compared to the GRU where the majority of fallers (68.3%) fell only once. In addition, a third (33.4%) of those who fell on the LSVU fell five or more times in comparison to the GRU where only 5.5% of the fallers sustained five or more falls.

**Table 1:** Characteristics of fallers on the GRU and LSVU

	**On GRU**	**On LSVU**	**Tests of significance**
*Gender:* count (%)			Pearson Chi-square = 40.26, 1df, *p* < 0.000
Male	65 (44.8%)	69 (88.5%)	
Female	80 (55.2%)	9 (11.5%)	
*Age*			
Mean age in years (SD)	82.0 (7.9)	87.4 (4.7)	T-test = −5.51 *p* < 0.000
*Falls:* count (%)			
Fell once	99 (68.3%)	23 (29.5%)	
Fell twice	22 (15.2%)	17 (21.8%)	
Fell 3 times	13 (9.0%)	8 (10.3%)	
Fell > 3 times	11 (7.5%)	30 (38.4%)	
Total number of falls	249	296	T-test = −4.94 p < 0.000
Mean number of falls per person (SD)	1.7 (1.5)	3.8 (3.5)	
*By gait aids listed at time of fall*			
No gait aids (cane, walker or wheelchair)	44 (30.3%)	30 (38.5%)	Pearson Chi-square = 28.41, 3df, *p* < 0.000
Cane only no walker or wheelchair	0 (0.0%)	5 (6.4%)	
Walker, with/without cane or wheelchair	95 (65.5%)	29 (37.2%)	
Wheelchair only, no cane or walker	6 (4.1%)	14 (17.9%)	
*Fallers by time of day of fall*			
Midnight to 3:59 a.m.	37 (25.5%)	13 (16.7%)	Pearson Chi-square = 15.0, 5df, *p* < 0.010
4:00 a.m. to 7:59 a.m.	25 (17.2%)	11 (14.1%)	
8:00 a.m. to 11:59 a.m.	17 (11.7%)	13 (16.7%)	
Noon to 3:59 p.m.	25 (17.2%)	7 (9.0%)	
4:00 p.m. to 7:59 p.m.	28 (19.3%)	14 (17.9%)	
8:00 p.m. to 11:59 p.m.	13 (9.0%)	20 (25.6%)	
*Fallers by injury sustained at time of fall*			Pearson Chi- square = 5.99, 1df, p < 0.014
Yes	57 (39.3%)	44 (56.4%)	
No	88 (60.7%)	34 (43.6%)	

*Note*: GRU: Geriatric Rehabilitation Unit; LSVU: long-stay Veterans’ Unit; SD: standard deviation.

As the people in these two settings significantly differed with respect to many key factors associated with repeat falling (e.g.: length of stay), further analyses comparing single fallers with repeat fallers were done by unit. For patients on the GRU, age reached statistical significance (Odds ratio: 1.05; 95% confidence interval: 1.000 – 1.102, p = 0.049) (see Table 2). Further, gender almost reached statistical significance (Odds ratio: 0.48; 95% confidence interval: 0.22-1.04, p = 0.06), suggesting that the odds of males being repeat fallers are 1/0.48 or 2.1 times the odds of females being repeat fallers on the GRU. However, among fallers on the LSVU, none of the study variables were significantly associated with repeat falls although there was some suggestion that time of day of first fall may be associated with repeat falls (see Table 3). The use of a walker was not associated with repeat falling on either unit.

**Table 2:** Factors associated with repeat fallers on GRU: logistic regression analysis

**Predictor variables**	**Odds ratio**	**Sig.**	**95% C.I.**	
			**Lower**	**Upper**
No gait aids (ref)	-	0.348	-	-
Walker with/without cane or wheelchair	0.615	0.244	0.272	1.392
Wheelchair only	0.271	0.272	0.027	2.776
Age	1.050	0.049	1.000	1.102
Gender (ref = male)	0.482	0.063	0.223	1.040
Injury	0.819	0.612	0.378	1.772
Midnight to 3.59 a.m. (ref)	-	0.886	-	-
4.00 a.m. to 7.59 a.m.	0.888	0.842	0.278	2.839
8.00 a.m. to 11.59 a.m.	0.670	0.566	0.171	2.629
Noon to 3.59 p.m.	0.768	0.664	0.234	2.525
4.00 p.m. to 7.59 p.m.	0.997	0.996	0.333	2.989
8.00 p.m. to 11.59 p.m.	1.802	0.398	0.460	7.065

ref = reference category.

95% C.I. = 95% confidence interval around the odds ratio.

GRU = Geriatric rehabilitation unit.

**Table 3:** Factors associated with repeat fallers on LSVU: logistic regression analysis

**Predictor variables**	**Odds ratio**	**Sig.**	**95% C.I.**	
			**Lower**	**Upper**
No gait aids (ref)	-	0.416	-	-
Cane only	3.921	0.320	0.266	57.835
Walker with/without cane or wheelchair	0.711	0.627	0.180	2.815
Wheelchair only	2.681	0.249	0.501	14.343
Age	1.002	0.979	0.884	1.135
Gender (ref = male)	0.639	0.615	0.111	3.666
Injury	1.039	0.948	0.325	3.323
Midnight to 3:59 a.m. (ref)	-	0.090	-	-
4:00 a.m. to 7:59 a.m.	13.052	0.019	1.538	110.765
8:00 a.m. to 11:59 a.m.	13.246	0.016	1.611	108.876
Noon to 3:59 p.m.	2.188	0.457	0.278	17.224
4:00 p.m. to 7:59 p.m.	6.143	0.068	0.874	43.150
8:00 p.m. to 11:59 p.m.	11.811	0.009	1.866	74.756

ref = reference category.

95% C.I. = 95% confidence interval around the odds ratio.

LSVU = Long-stay veterans’ unit.

Sig: Level of significance.

## Discussion

This study has attempted to compare two quite different types of geriatric units in order to explore the issue of repeat fallers in two such different settings. Given that fall prevention strategies were already in place in both settings, this study may provide some insight into how to focus a secondary wave of fall prevention in different settings.

Factors associated with repeat falls differed by unit. Among fallers on the GRU, only age reached statistical significance for repeat falls but only at the 0.05 level, which given the large number of comparisons made, needs to be viewed with caution. The GRU may be seen as a very high risk environment, given that many of the patients have been admitted from an acute care hospital and most are deconditioned as well as carrying the burden of their recent illness. This is reflected in their high rate of first falls. For such a population the adoption of universal prevention measures is indicated. However, falls and repeat falls do occur and the risk of repeat falling appears to be greatest in the very old. Further fall prevention strategies may need to be specifically designed for, and more focused on, the older GRU patients, who are likely to be frailer and possibly sicker. A study of the factors operating in the very old may need to be undertaken to identify potentially modifiable factors to guide more specific interventions in these people.

Among fallers on the LSVU, none of the study variables were significantly associated with repeat falls. However, it is noteworthy that while falling on the LSVU was less common, repeat falling was more so. These patients are medically more stable and likely more independently mobile. Under these circumstances, residents who fall despite the presence of generic prevention programs, seem to identify themselves as being at high risk for repeat falling. One question raised by these results is whether a more generic falls prevention program is likely to be sufficient in such a setting or whether, in such a setting, individualized efforts should focus on first time fallers, with the goal being to determine the cause of the fall in order to reduce the risk of another fall. In a way one is relying on the patients to declare themselves at risk by falling once, and they can potentially be seen as people for whom the generic programs are insufficient. Of some reassurance is the observation that the risk of serious injury in the first fall was low.

The present study did not find any association between having a walker listed as a gait aid on the Incident Reporting Tool at time of first fall and repeat falling. However, it could not be determined if the fall was sustained while actually using a walker or if the fall event occurred in a patient who usually uses a walker but was not using their walker as prescribed. In a previous study it was shown that the use of a walker may be an added risk factor for falling in people living alone, the assumption being that if you live alone you may have to perform activities yourself for which a walker is a hazard [18]. Whether similar situations exist in these care settings is not known. This uncertainty points to the need for further research on the use/non-use of the mobility aid at the time of the fall and the associated patient activities.

This study suggests that some repeat fall prevention strategies should be specific to the person (e.g., greater attention to older GRU patients), some strategies should be unit (population) specific (e.g., higher risks at certain times of day on the LSVU) and these are in addition to global strategies that are in place to enhance safety and reduce falls risk across the institutional setting (e.g. assessment and incident recording protocols). Strategies that include a comprehensive fall risk assessment which focused on the common and recognized extrinsic and intrinsic risk factors for all persons at time of admission, as suggested by the Canadian Task Force on Preventive Health Care, may reduce falls and perhaps also repeat falls. The impact of such a strategy has already been demonstrated in randomized control trials conducted in LTC settings [19] and in sub-acute rehabilitation settings [20].

There is emerging evidence that fall risk profiles and evidence-based approaches to intervention differ considerably among different geriatric care settings [14,21,22]. These differences in risk factors and risk profiles may be attributable to differences in the type of setting, the measurement tools used, the population demographics and characteristics with a few risk factors inherent for that particular setting [23].

Given that our study was conducted in units where fall prevention programs are in place, but falls still occur, this may speak to the need for a secondary phase of interventions at a more individual level directed to those for whom the global interventions are insufficient. The identification of factors which might predict further falling in those who have fallen once may allow more focused attention on those so identified in order to reduce future risk.

### Study limitations

The present study has several limitations. Data from the pilot version of the Incident Reporting Tool were used to identify factors associated with repeat fall events. However, some of the data elements were not included or were incorrectly coded in this new tool. Level of incident (near-miss or actual fall event) was not consistently captured and gender of the patient/resident who fell, a factor known to be associated with repeat falls, was not included. Individual-specific dates of admission and discharge were also not available, thereby limiting assessment of exposure to fall risk (i.e. length of stay). Such data need to be added later. In addition, retrospective analysis of data using incident reports has been known to be confounded by issues of partial recording and under-reporting and by the fact that such tools were not designed specifically for research. However, they are a valuable source of information as they can be examined to identify population specific risk factors for falls, which can be utilized for targeted fall prevention strategies [4].

As well it was challenging to interpret some of the information. For example, it was not clear if the person was actually using the walker as a gait aid when s/he experienced the fall. While there is a team debrief following each fall event that likely captures a number of contextual factors, this information is not recorded in this database. Variables shown by the literature as being key to understanding repeat fall events such as mobility deficits, cognitive issues, and medications taken within the last 24 hours were not included in this version of the database thereby limiting a greater exploration of relationships among the predictor variables. Moreover, interpretation is difficult without control data drawn from the non-fallers on the units.

Finally, despite using two years of data, unit specific analyses lacked power.

## Conclusions

This study attempted to identify patterns among repeat fallers in diverse geriatric patient populations. However, this goal was met with only modest success. Among fallers on the GRU, only age reached statistical significance for repeat falls. Further, on the LSVU, none of the study variables were significantly associated with repeat falls, although time of day may be worth further exploration. Better information collection with a specific focus on the issue of repeat falls may be more successful in identifying the fall-related risk factors and subsequently developing targeted interventions.

Further information on the use of mobility aids and repeat falls is needed. Prospective studies could be conducted in different geriatric care settings to compare people who were using gait aids at the time of fall with those not using them, including those who should have been using them and those for whom they were not prescribed.

## Competing interests

The authors declare that they have no competing interests.

## Authors’ contributions

HT carried out the study and participated in conceptualization of the study, acquisition of data, analysis and interpretation of data and drafting the manuscript. MK supervised the carrying out of the study and participated in study conceptualization, design, coordination, data interpretation and manuscript revision. RGC participated in study conceptualization, design, data interpretation and manuscript revision. IG participated in study conceptualization, design, performed data analysis and interpretation and participated in manuscript revision. MG participated in study design, data interpretation and manuscript revision. All authors read and approved the final manuscript.

## Pre-publication history

The pre-publication history for this paper can be accessed here:

http://www.biomedcentral.com/1471-2318/13/106/prepub

## References

[B1] Scott V Wagar B Sum A Metcalfe S Wagar L A public health approach to fall prevention among older persons in Canada Clin Geriatr Med 2010 26 705 718 10.1016/j.cger.2010.06.003 20934617

[B2] American Geriatrics Society, British Geriatrics Society, and American Academy of Orthopaedic Surgeons Panel on Falls Prevention Guideline for the prevention of falls in older persons J Am Geriatr Soc 2001 49 664 672 10.1046/j.1532-5415.2001.49115.x 11380764

[B3] Public Health Agency of Canada Report on seniors’ falls in Canada 2005 http://www.phac-aspc.gc.ca/seniors-aines/alt

[B4] Oliver D Preventing falls and falls-injuries in hospitals and long-term care facilities Rev Clin Gerontol 2007 17 75 91

[B5] Aronow W Ahn C Association of postprandial hypotension with incidence of falls, syncope, coronary events, stroke, and total mortality at 29-month follow-up in 499 older nursing home residents J Am Geriatr Soc 1997 45 1051 1053 9288010 10.1111/j.1532-5415.1997.tb05965.x

[B6] Kiely D Kiel D Burrows A Lipsitz L Identifying nursing home residents at risk of falling J Am Geriatr Soc 1998 4 551 555 9588366 10.1111/j.1532-5415.1998.tb01069.x

[B7] Ryynänen O-P Kivelä S-L Honkanen R Laippala P Recurrent elderly fallers Scand J Prim Health Care 1992 10 277 283 10.3109/02813439209014074 1480867

[B8] Lipsitz LA Jonsson PV Kelley MM Koestner JS Causes and correlates of recurrent falls in ambulatory frail elderly J Gerontol 1991 46 M114 M122 10.1093/geronj/46.4.M114 2071832

[B9] Bateni H Maki BE Assistive devices for balance and mobility: benefits, demands, and adverse consequences Arch Phys Med Rehabil 2005 86 134 145 10.1016/j.apmr.2004.04.023 15641004

[B10] Tideiksaar R Falls in Older People: Prevention & Management 2010 Baltimore: Health Professions Press

[B11] Brown CJ Norris M Falls: physician’s information and education resource (PIER) American College of Physicianshttp://pier.acponline.org/physicians/screening/s168/pdf/s168.pdf

[B12] Ontario Injury Prevention Resource Centre Wheelchair or adult walker falls: Ontario injury compass http://www.oninjuryresources.ca/downloads/Compass/2007/2007-03-OICompass-Wheelwalk.pdf

[B13] Registered Nurses Association of Ontario Nursing best practice guidelines: Prevention of falls and fall injuries in the older adult 2005 http://www.rnao.ca/bpg/guidelines/prevention-falls-and-fall-injuries-older-adult

[B14] Nyberg L Gustafson Y Janson A Sandman P Eriksson S Incidence of falls in three different types of geriatric care Scand J Public Health 1997 25 8 13 10.1177/140349489702500103 9106939

[B15] Norris MA Walton RE Patterson CJS Feightner JW Canadian Task Force on Preventive Health Care Prevention of Falls in Long-Term Care Facilities: Systematic Review and Recommendations 2003 London: Canadian Task Force

[B16] Ontario Ministry of Health and Long-Term Care Long-Term Care Homes Act 2007 http://www.e-laws.gov.on.ca/html/source/regs/english/2010/elaws_src_regs_r10079_e.htm

[B17] Hosmer DW Jr Lemeshow S Applied Logistic Regression 2000 2 New York: John Wiley & Sons

[B18] Crilly RG Lytwynec S Kloseck M Smith JM Olsen T Gold B Patient outcomes after discharge from a geriatric day hospital Can J Aging 2005 24 3 305 310 10.1353/cja.2005.0076 16421854

[B19] Becker C Kron M Lindemann U Sturm E Eichner B Walter-Jung B Effectiveness of a multifaceted intervention on falls in nursing home residents J Am Geriatr Soc 2003 51 306 313 10.1046/j.1532-5415.2003.51103.x 12588573

[B20] Haines TP Bennell KL Osborne RH Hill KD Effectiveness of a targeted falls prevention program in a subacute hospital setting: a randomised controlled trial Br Med J 2004 328 676 10.1136/bmj.328.7441.676 15031238 PMC381222

[B21] Jensen J Lundin-Olsson L Nyberg L Gustafson Y Fall and injury prevention in older people living in residential care facilities: a cluster randomized trial Ann Intern Med 2002 136 733 741 10.7326/0003-4819-136-10-200205210-00008 12020141

[B22] Scott V Votova K Scanlan A Close J Multifactorial and functional mobility assessment tools for fall risk among older adults in community, home-support, long-term and acute care settings Age Ageing 2007 36 130 139 10.1093/ageing/afl165 17293604

[B23] Morrison G Lee HL Kuys SS Clarke J Bew P Haines TP Changes in falls risk factors for geriatric diagnostic groups across inpatient, outpatient and domiciliary rehabilitation settings Disabil Rehabil 2011 33 900 907 10.3109/09638288.2010.514019 21446880

